# Progress in Surgery

**Published:** 1898-07-02

**Authors:** 


### Progress in Surgery.

### CEREBRAL SURGERY.

**Technique.**—It would appear that prospective advances in cerebral surgery will be in the directions of improved methods of diagnosis and perfected operative details. By means of the former the cases subjected to operation will be more carefully selected, and by attention to the latter the risks from hæmorrhage and sepsis will be diminished. In a recent paper Tiffany[1] summarises his practice as regards the carrying out of an intra-cranial operation. It does not materially differ from that of Horsley, Macewen, and others of wide experience in this field of surgery; but there are one or two points worth drawing attention to. While he personally prefers to open the skull with chisel and mallet, he says that each surgeon should select the instrument with which he is most familiar, and in the majority of cases this is the trephine. E. Cotterell[2] believes that the modern pattern of trephine is, all round, the best instrument for this purpose, further removal of bone, if necessary, being effected with rongeur forceps. Tiffany has had no satisfaction in using a tourniquet round the head to control hæmorrhage from the scalp. Cotterell, on the other hand, is satisfied that an ordinary silk elastic band, one inch wide, wound round on a level with the occiput and forehead five or six times, is of great value. He has done many operations on children with this tourniquet without losing half a drachm of blood, and without requiring to ligature any vessels of the scalp. For parenchymatous bleeding from the brain Tiffany trusts to gauze pressure. He always employs a drain for some time.

There has been a tendency lately to give up the practice of replacing trephine circles after closing the dura, it having been found that these often either necrose or become absorbed. An important and valuable series of experiments on this point has lately been made by David,[3] who finds that these reunite when implanted aseptically just like other tissues. His experiments were done on dogs, and the animals were killed at regular intervals, so that the process of repair could be closely observed. Macewen's original clinical observations are thus confirmed.

**Drainage for Hydrocephalus.**—Several attempts have recently been made to influence the progress and alleviate the symptoms resulting from internal hydrocephalus. In a case reported by Bruce and Stiles, of Edinburgh,[4] the fourth ventricle was opened and drained for acquired hydrocephalus in a girl, aged thirteen, who had inherited syphilis. Headache, with retraction of the head and pain in the back, were associated with marked emaciation in spite of a ravenous appetite. Various eye symptoms, almost complete paralysis of the limbs, and paralytic distension of the bladder ensued. Pulse 102 to 120, temperature irregular, reaching 103 deg. F. There was no vomiting. Mr. Stiles removed a trephine circle, three-quarters of an inch in diameter, and exposed the membranes, on opening which about ten ounces of cerebro-spinal fluid escaped from the fourth ventricle. Great quantities of cerebro-spinal fluid continued to escape, and the general condition improved for some time, but the patient died three weeks later. Post-mortem chronic basal syphilitic meningitis was found, as well as an abscess in the kidney.

G. A. Sutherland and Watson Cheyne[5] report a case in which operation was performed in a congenitally syphilitic child of six months with hydrocephalus. A catgut drain was passed through an opening on the left lower angle of the anterior fontanelle, through the brain into the lateral ventricle. Although very little fluid escaped a steady and marked diminution in the size of the skull took place. Three months later, however, the child died from basilar meningitis. Cheyne showed another case where similar diminution in the size of the skull resulted.

**Cerebral Tumours.**—At the Moscow Congress Bergmann[6] sounded a note of warning against indiscriminate operating for cerebral tumours. He believes that cases in which operation is likely to do good are few in number. Exploratory operations he specially condemns. In the majority of these no tumour was found, while the risks of the operation are considerable from bleeding, shock, epilepsy, and hernia cerebri. It is only when the tumour occupies the motor area and gives definite evidence of its site that operations are hopeful.

In discussing the difficulties of locating cerebral tumours L. Bruns[7] expresses his belief in the value of percussion of the skull, as suggested by Macewen, at least as a confirmatory sign.

Obici and Bollici[8] state that by the aid of the Roentgen rays they have been able to localise brain tumours. Jolly entirely failed to do so.

**Hydatid of Brain.**—A case of successful operation for hydatid cyst of the brain is reported by O'Hara,[9] of Melbourne. Symptoms pointed to the left motor area as the seat of lesion, and on exposing this a cyst containing clear fluid was found, emptied and drained. Recovery with improvement in symptoms followed. No hooklets were found in the fluid, which contained an excess of sodium chloride.

**Epilepsy.**—Curtis,[10] in reporting several cases of operations for epilepsy, makes some general remarks on the subject. Before operation is undertaken accurate localisation and a definite form of lesion must be assured. Indiscriminate operating will result in such disappointment and discredit that it will be impossible to obtain permission to operate even in suitable cases. Cabot[11] reports a case partially successful.

**Trephining for Idiocy.**—The results of this operation have so far not been encouraging. Curtis reports two cases in which operation did no good, although the patients recovered well from the operation. Griffiths[12] divides cases of microcephalus into two classes—(*a*) those with small, ill-filled, but not deformed skulls, and (*b*) those with small and deformed skulls. He does not think operation can be of any use in either class, except, perhaps, to alleviate serious motor symptoms. The operation mortality has been very high hitherto. Among those who discussed this paper there was a general consensus of opinion against this operation.

[1] Annals of Surgery, 1897. [2] Clinical Journal, Dec., 1897. [3] Arch. für Klin. Chir., Bd. liii., Heft. 4. [4] Lancet, Jan. 29, 1898. [5] Brit. Med. Jour., Mar. 19, 1898. [6] Brit. Med. Jour. Epit., Dec. 4, 1897. [7] Brit. Med. Jour. Epit., Feb. 5, 1898. [8] Brit. Med. Jour. Epit., Mar. 12, 1898. [9] Australian Med. Gaz., Feb 21, 1898. [10] Post Graduate, Mar., 1898. [11] Boston Med. and Surg. Jour., June 3, 1897. [12] Lancet, Mar. 12, 1898.

### SURGERY OF THE SPLEEN AND PANCREAS.

**Splenectomy.**—Martyn Jordan[1] favours a partial excision where possible. He has experimented upon 22 dogs by his conservative method, with 21 recoveries. Jonnesco,[2] as the result of his experience, recommends that the surgeon should stand on the patient's right side, because from this side he obtains a better view of the pedicle, the ligation of which is the most difficult and important step in the operation. (1) The abdominal incision is best made in the middle line, beginning at the ensiform cartilage, and extending to or below the umbilicus. (2) The spleen is then isolated. This varies in difficulty according to the extent of adhesions or laxity of the pedicle. Extensive and firm adhesions may be a contra-indication to continuing the operation. To reach and examine the phreno-splenic ligament the operator, covering the spleen with a gauze pad, pulls it to the right, while an assistant draws the left lip of the abdominal wound to the left. This exposes the bed of the spleen and the diaphragmatic vault. Having divided the phreno-splenic ligament, the adhesions of the spleen to its bed are next attacked. (3) Section of the pedicle. When delivered from the abdominal cavity, the spleen is turned over to the left on its convex surface, so as better to expose the internal surface and its pedicle. Division of the pedicle ought to be accomplished by separating and dividing, between two ligatures, each vessel in turn from the lower side upwards. The gastro-splenic omentum is a continuation of the pedicle, and should be treated in a similar fashion.

(4) Revision of the splenic bed and final hæmostasis is the next step in the operation. (5) Closure of the abdominal wound, and application of dressings. The author has collected 36 cases of splenectomy for malarial enlargement, with a death-rate of 50 per cent. From 1887 to 1896 the death-rate was 31·7 per cent.; from 1891 to 1896 the death-rate was 15·4 per cent., the improvement doubtless being due to asepsis, and a careful choice of cases. Among the contra-indications may be mentioned: (1) Profound cachexia, (2) extensive adhesions, (3) size of the spleen, (4) leucocythæmia. The author believes it useless and dangerous to prolong medical treatment, because as long as the spleen is hypertrophied from malaria cachexia increases, and therefore the chances of cure by operation decrease. Bernhard[3] relates a successful case of splenectomy for rupture of the spleen without external wound. Some 160 cases of splenectomy have been reported for various conditions. In 28 cases the spleen was ruptured and prolapsed into the wound, and the results of the splenectomy here were favourable. There are only six cases on record in which the spleen has been extirpated for rupture without external wound, and five of these died.

**Cyst of the Pancreas.**—Doran[4] says these cysts may occur at any age. They may lie in the lesser peritoneal cavity, or between the folds of the transverse meso-colon, or in the general peritoneal cavity. When it occupies the lesser peritoneal cavity it has probably burst through the peritoneum in front of the pancreas. This would account for the complete freedom of the ascending layer of the transverse meso-colon, which is found entirely below the cyst in many of these cases. When a pedicle can be formed, even if it include the tail of the pancreas itself, total removal of the cyst is justifiable. Removal of a sessile cyst, on the other hand, involved risk of damage to the splenic and other large vessels, and to small arteries and veins in the friable pancreatic tissue. The statistics of drainage in the treatment of sessile cysts are very satisfactory. Aspiration and tapping for diagnostic purposes are both perilous, and seeking for a calculus during the operation is not without danger. Out of about 70 published cases of incision and drainage only five died. Turner[5] mentions a case of pancreatic cyst, and Rolleston and Turner[6] contribute a case of peri-pancreatic cyst. Malcolm[7] records a successful case of complete removal of a multilocular cyst of the pancreas. During the operation much of the fluid was withdrawn by aspiration from two of the chief loculi; that from one was quite clear, that from the other blood-stained. The ropy, dull-brown mixture of the two was found to be strongly amylolytic when tested on starch solution. The author thinks that when a pancreatic cyst has been diagnosed and drainage decided on, the opening into the cyst should be made from the loin, if the tumour presents in that position on either side. But the diagnosis should first be verified by abdominal section. Drainage through the loin is easy, and as the cyst contracts it naturally falls backwards and closes on the opening, whereas if the cyst be attached to the lips of the incision in the anterior abdominal wall, and drained thus, it must form a long, and perhaps a funnel-shaped, tube across the abdomen, difficult to drain, and possibly a source of other troubles as it contracts.

[1] Lancet, Jan. 22, 1898, p. 208. [2] Annals of Surgery, Dec., 1897, p. 723. [3] Brit. Med. Jour. Epit., Dec. 25, 1897. [4] Lancet, Dec. 18, 1897, p. 1,591, and Brit. Med. Jour., Dec. 18, 1897, p. 1,779. [5] Lancet, Dec. 18, 1897, p. 1,591. [6] Ibid. [7] Ibid., Jan. 29, 1898, p. 279.

